# Predictors for bile duct stone recurrence after endoscopic extraction for naïve major duodenal papilla: A cohort study

**DOI:** 10.1371/journal.pone.0180536

**Published:** 2017-07-10

**Authors:** Shin Kato, Kenji Chinen, Susumu Shinoura, Kaoru Kikuchi

**Affiliations:** Department of Gastroenterology, Okinawa Prefectural Chubu Hospital, Uruma, Okinawa, Japan; Hokkaido University, JAPAN

## Abstract

**Background:**

Predictors for bile duct stone recurrence after endoscopic stone extraction have not yet been clearly defined and a study investigating naïve major duodenal papilla is warranted because studies focusing only on naïve major duodenal papilla are rare. The aim of this study was to observe the long-term outcomes of endoscopic bile duct stone extraction for naïve major duodenal papilla and to assess the predictors for recurrence.

**Methods:**

This was a retrospective cohort study that consisted of 384 patients with naïve papilla who underwent initial endoscopic bile duct stone extraction. Patients were followed up in outpatient department subsequent to complete stone clearance. Recurrence was defined as symptomatic repeated stone formation observed at least three months after the procedure. Stone recurrence, predictors of recurrence, and the recurrence rate, depending on each endoscopic treatment for major duodenal papilla, were examined.

**Results:**

In this study, 34 patients (8.9%) developed stone recurrence. The median time to recurrence was 439 days. Periampullary diverticulum and multiple stones were strong predictors of bile duct stone recurrence (RR, 5.065; 95% CI, 2.435–10.539 and RR: 2.4401; 95% CI: 1.0946–5.4396, respectively). The above two factors were independent predictors of stone recurrence as per logistic regression analysis adjusted for confounders (Periampullary diverticulum: OR, 7.768; 95% CI, 3.27–18.471; multiple stones: OR, 4.144; 95% CI, 1.33–12.915). No recurrence was observed after endoscopic papillary large balloon dilatation (0/20), whereas recurrence was observed in 7 patients after endoscopic papillary balloon dilatation (7/45) and in 27 patients after endoscopic sphincterotomy (27/319). However, these differences were not statistically significant (p = 0.105).

**Conclusions:**

We determined that the presence of periampullary diverticulum and multiple stones are strong predictors for recurrence after endoscopic stone extraction. Moreover, endoscopic papillary large balloon dilatation tended to be correlated with non-recurrence of bile duct stone.

## Introduction

Techniques for endoscopic bile duct (BD) stone extraction are well established and efficacious, and the complete clearance rate of ordinary sized BD stones is approximately 92%–100% [1, 2]. However, recurrence of BD stones occurs in approximately 10% of patients after endoscopic stone extraction.

Suggested predictors of BD stone recurrence after endoscopic stone extraction include dilated BD, large stones, multiple stones, and periampullary diverticulum (PAD). However, predictors for BD stone recurrence have not yet been clearly defined because of variability in study designs.

To note, a study investigating naïve major duodenal papilla is warranted given that the recurrence rate of BD stones is clearly elevated in patients with a history of BD stone extraction [3]. However, studies focusing only on naïve major duodenal papilla are rare. In addition, the relationship between the type of treatment for major duodenal papilla before stone extraction and recurrence is not well known.

The aim of this study was to observe retrospectively the long-term outcomes of endoscopic BD stone extraction for naïve major duodenal papilla. The predictors for BD stone recurrence and correlation of the type of treatment for major duodenal papilla and stone recurrence were assessed.

## Methods

**Fig 1:**
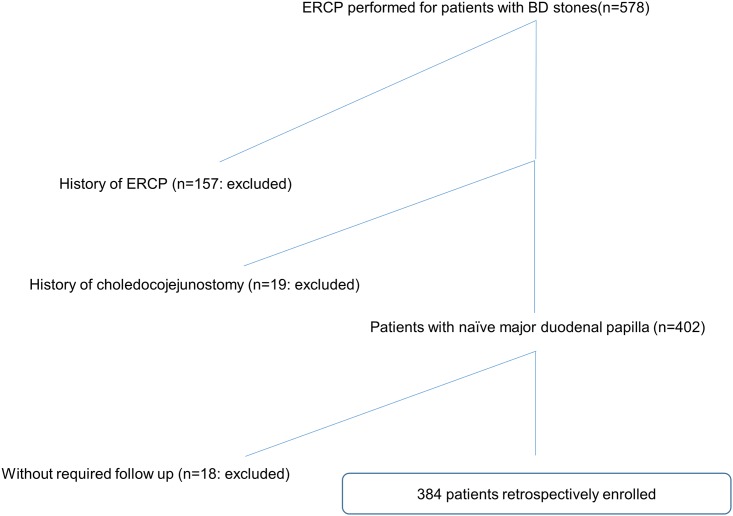
Enrollment flowchart. Among 578 patients who underwent ERCP for BD stone extraction, 194 patients were excluded and finally 384 patients were enrolled.

From January 2009 to November 2014, 578 consecutive patients underwent endoscopic retrograde cholangiopancreatography (ERCP) for BD stone extraction in a 550 beds, tertiary referral center located in Japan. Among them, 157 patients were excluded because of previous ERCP history, and 19 patients were excluded because of previous history of choledocojejunostomy. While 402 patients had initial endoscopic BD stone extraction for naïve major duodenal papilla, 13 of them did not have regular follow up after stone extraction and were therefore excluded. In addition, five patients could not complete the required follow-up period (3 months) because of death from primary disease (pancreatic cancer, 1; lung cancer, 1; malignant lymphoma, 1; and urosepsis, 2). Thus, 384 patients were included in this retrospective, observational study (Fig 1).

After obtaining written informed consent for ERCP, all procedures were performed under moderate sedation using a JF 260V or TJF 260V duodenal endoscope (Olympus Medical Systems Co. Ltd, Tokyo, Japan). Staff endoscopists with over 10 years of experience of performing ERCP performed or supervised all procedures. Treatment for major duodenal papilla was performed using CleverCut sphincterotome (Olympus Medical Systems Co. Ltd.) or Autotome TM sphincterotome (Boston Scientific Japan, Tokyo, Japan) for endoscopic sphincterotomy (ES), Hurricane TM dilatation balloon (6 mm, 8 mm, and 10 mm, Boston Scientific Japan) for endoscopic papillary balloon dilatation (EPBD), and CRE dilatation balloon (maximum diameters: 12 mm, 15 mm, and 18 mm, Boston Scientific Japan) or Giga balloon (Century Medical, Inc. Tokyo, Japan) for endoscopic papillary large balloon dilatation (EPLBD). ES was generally performed by adding a middle-length incision (completely cutting over the first hooding fold). In EPBD, we inflated the balloon until disappearance of the waist of the balloon and held inflation for 15 s. In cases treated using EPLBD, small-length ES was added before dilatation. Dilating balloon diameter was determined depend on the basis of the distal bile duct size. After inflation and disappearance of the balloon waist, the balloon was rapidly deflated. Stones were extracted via balloon catheter or basket catheter. We confirmed stone clearance by double-contrast fluoroscopy using balloon catheter or intra-ductal ultrasonography subsequent to completing endoscopic stone extraction.

The number and diameter of stones and BD diameter were confirmed via fluoroscopic images. In this study, muddy stones or sludge, which was recognized as solid stones on fluoroscopic images, was classified as stone and recorded as sludge. The presence of PAD was confirmed via images of the duodenoscopy. The presence of calculus gall bladder (GB) before stone extraction was confirmed using images at the time of admission. History of cholecystectomy during follow up period was recorded.

All patients were discharged and underwent follow-up after procedure in the outpatient department (the initial follow-up was scheduled within 4 weeks of discharge, and after that, every 2–3 months). Interview for clinical symptoms and laboratory testing (including liver function test and complete blood count) were performed in the outpatient department. Imaging work up (abdominal ultrasonography or computed tomography) was performed in cases with clinical symptoms or abnormal laboratory findings while checking for recurrence. We retrospectively analyzed patient characteristics; stone recurrence; predictors of recurrence, periampullary diverticulum (PAD), presence of the calculus gall bladder (GB), BD dilatation, large stones, multiple stones, required several times procedure for complete stone extraction, difficult BD cannulation, using endoscopic mechanical lithotripsy (EML); and recurrence rate, which depended on treatment type.

### Definitions

Naïve major duodenal papilla was defined as papilla Vater without any treatment history including ES, EPBD, EPLBD, and biliary stenting. Difficult BD cannulation was defined as a procedure that necessitated more than 20 minutes for BD cannulation or pre-cutting. Recurrence was defined as repeated stone formation with clinical signs or symptoms observed at least 3 months after the initial procedure.

### Statistical analysis

Statistical analysis was performed via StatMate 4 (ATMS Co. Ltd, Tokyo, Japan). The chi-square test or Fisher’s exact test was used to analyze categorical data. To evaluate the recurrence rate depend on each treatment, the Marasculio procedure was performed. The relative risk was calculated for each predictor of BD stone recurrence. Kaplan Meier curve analysis was performed along with the log-rank test to assess patient recurrence free survival. Logistic regression analysis was also used to adjust for potential confounders. Differences with a *p* value of < 0.05 were considered statistically significant.

## Results

There were 200 men and 184 women in this study, and the mean age was 70.8 years old. The median follow up period was 1098 days (ES, 1160 days; EPBD, 1216 days; EPLBD, 789 days. range, 92–2552 days). Additionally, 136 patients (35.4%) had PAD, and 10 patients had type 1 PAD (in which the major duodenal papilla is located inside the diverticulum), 33 patients had type 2 (major duodenal papilla is located on the edge of the diverticulum), 91 patients had type 3 (major duodenal papilla is located on the outside of the diverticulum), and 2 patients had unknown/undetermined type of PAD due to lack of records. In our cohort, 319 patients had ES before stone extraction, 45 patients had EPBD, and 20 patients had EPLBD. Mean diameters of stone and BD were 7.4 mm (range, 3–35 mm), and 10.3mm (range, 4–25 mm), respectively. Procedural mean time was 42.9 min (range 7–158 min). The number of BD stones were as follows; 1 stone, 212 patients; 2 stones, 39 patients; 3 stones, 28 patients; 4 stones, 15 patients; 5 stones, 7 patients; 6 stones, 12 patients; 7 stones, 8 patients; 8 stones, 4 patients; sludge, 59 patients. (Table 1).

**Table 1:** Patients characteristics.

**Sex**	Man:200, Woman 184
**Age**	2–101 (mean. 70.8)
**ECOG-PS**	PS0:174, PS1:88, PS2:64, PS3:41, PS4:17
**Follow up period (day)**	92–2552 (median 1098)
**PAD**	136 (35.4%). type I:10, typeII:33, typeIII:91,unknown:2
**Treatment for papila**	ES:319, EPBD:45, EPLBD:20.
**Diameter (mm)**	Stone 3–35 (mean.7.4), Bile duct 4–25 (mean.10.3)
**Procedure time (min.)**	7–158 (mean 42.9)
**Number of stones**	<1>:212, <2>:39, <3>:28, <4>:15, <5>7, <6>:12, <7>:8, <8>:4, <sludge>:59

PS, performance status; PAD, periampullary diverticulum; ES, endoscopic sphincterotomy; EPBD, endoscopic papillary balloon dilatation; EPLBD, endoscopic papillary large balloon dilataion.

Among 384 patients, 34 patients (8.9%) developed BD stone recurrence during the follow up period. The median time to recurrence was 439 days (range 92–1510 days).

Stone recurrence was observed four times in 1 patient, two times in 2 patients, and one time in 31 patients.

Table 2 shows the analytical results for predictors of BD stone recurrence. PAD was observed in 25 cases among the 34 patients (73.5%) with BD stone recurrence, while PAD was observed in 111 cases among 350 patients (31.7%) without BD stone recurrence (RR, 5.065; 95% CI, 2.435–10.539). PAD types, especially type 1 PAD, significantly correlate with BD stone recurrence (4 patients with recurrence vs. 6 patients without recurrence, *p* < 0.01). The existence of multiple BD stones (≥5) was also detected as a strong predictor. Multiple stones were observed in 6 cases among cases with recurrence and observed in 25 cases among cases without recurrence (RR: 2.4401; 95% CI: 1.0946–5.4396). Presence of calculus GB, dilated BD, large stones (≥ 10 mm), using EML were not significant predictors. Logistic regression analysis, adjusted for confounders, revealed that the existence of PAD and multiple stones were independent predictors of BD stone recurrence (PAD: OR, 7.768; 95% CI, 3.27–18.471 and multiple stones: OR, 4.542; 95% CI, 1.49–13.898) (Table 3).

**Table 2:** Relative risk for stone recurrence.

	Recurrence +	Recurrence -	Relative risk	95% CI
Sex (Man)	20	180	1.3143	0.6841≤RR≤2.5251
Age (≥ 80)	10	96	1.0927	0.5410≤RR≤2.2071
**PAD**	**25**	**111**	**5.0654**	**2.4345≤RR≤10.5389**
Presence of GB	9	90	1.0364	0.5010≤RR≤2.1437
BD dilatation (≥15mm)	5	34	1.5252	0.6267≤RR≤3.7121
Large stone (≥ 10mm)	8	51	1.6949	0.8068≤RR≤3.5606
**Multiple stones (≥ 5)**	**6**	**25**	**2.4401**	**1.0946≤RR≤5.4396**
Several procedure	5	26	1.9633	0.8183≤RR≤4.7107
Difficult BD cannulation	2	17	1.2007	0.3106≤RR≤4.6411
EML	5	36	1.4424	0.5911≤RR≤3.5199

PAD, periampullary diverticulum; GB, gall bladder; BD, bile duct; EML, endoscopic mechanical lithotripsy.

**Table 3:** Multivariate analysis for stone recurrence (Logistic regression test).

	Odds ratio	95% CI	*p* value
**PAD**	**7.7676**	**3.2666≤OR≤18.4707**	**<0.01**
Presence of GB	1.6870	0.7113≤OR≤4.0012	0.235
BD dilatation (≥15mm)	1.5035	0.4716≤OR≤4.7927	0.491
Large stone (≥ 10mm)	0.8693	0.2636≤OR≤2.8662	0.817
**Multiple stone (≥ 5)**	**4.1436**	**1.3296≤OR≤12.9152**	**0.014**
EML	1.2973	0.3255≤OR≤5.1711	0.712

PAD, periampullary diverticulum; GB, gall bladder; BD, bile duct; EML, endoscopic mechanical lithotripsy.

**Fig 2:**
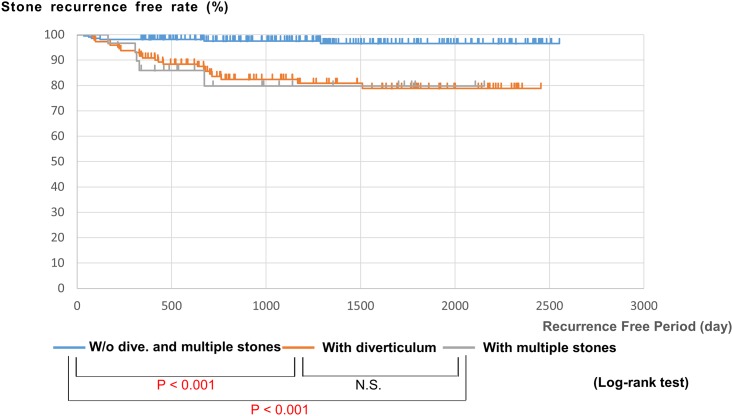
Recurrent free period depend on the group with or without significant predictor (Kaplan Meier curve analysis and log-rank test). Group with PAD or multiple stones showed significantly short period until recurrence compared to the group without those predictors (p<0.001, respectively).

Fig 2 demonstrates the Kaplan–Meier analysis for the recurrence free period in patients with or without the significant predictors. The patients groups with PAD or multiple stones showed a significantly shorter period until recurrence compared to the patient group without these predictors (*p* < 0.001).

The recurrence rate was not distinct regarding the treatment type for major duodenal papilla (Table 4). Seven cases of recurrence were observed in patients after EPBD (7/38), 27 cases of recurrence in the patients after ES (27/292), while 0 case of recurrence in patients after EPLBD (0/20) (*p* = 0.105).

**Table 4:** The recurrence rate depended on the each treatment (Marasculio procedure).

	Recurrenced	Not recurrenced	*p* value
**EPLBD**	0	20	0.105
**EPBD**	7	38	
**ES**	27	292	

EPLBD, endoscopic papillary large balloon dilataion; EPBD, endoscopic papillary balloon dilatation; ES, endoscopic sphincterotomy.

## Discussion

In this study, we found that the recurrence rate of BD stones during follow up period was 8.9%, and the presence of PAD and multiple BD stones were strong predictors of recurrence.

Several previous studies performed long term follow up after endoscopic BD stone extraction with treatment for major duodenal papilla, including non-naïve papilla cases. While several reports described stone recurrence and its predictors after stone extraction with ES, [4–10] it was unclear if all patients had no previous treatment for papilla. Few reports have mentioned stone recurrence after endoscopic BD stone extraction for naïve major duodenal papilla treated via not only ES but also EPBD. Yasuda et al. reported the long-term follow-up results after endoscopic stone extraction using ES or EPBD and demonstrated that several factors (PAD, ES, and *in situ* gall bladder stones) resulted in stone recurrence. [11] Our study potentially provides a unique insight like the abovementioned report given only naïve major duodenal papilla cases were enrolled. In addition, ES was not the only treatment for major duodenal papilla investigated in our work, and we analyzed multiple recurrence predictors.

Several other reports indicated the recurrence rate as between 9.7% and 15%[4–8, 12–16], mainly around 10%. In our study, the recurrence rate was 8.9%, comparable to previous mentioned studies. Although the 10% recurrence rate is common, Sugiyama et al. mentioned a 30.9% recurrence rate after endoscopic BD stone extraction in patients with a previous history of recurrence. [3] This work indicated that the risk of recurrence could elevate in patients with a previous history of such as compared to cases of initial endoscopic stone extraction.

As earlier noted, PAD has been reported as a predictor. Pereira-Lima et al. reviewed 203 post-ES patients and concluded that the presence of PAD is a strong predictor of BD stone recurrence after endoscopic stone extraction similarly to BD dilatation larger than 15 mm. [7] Keizmann et al. analyzed 45 elderly patients after endoscopic stone extraction compared with 51 younger patients. They mentioned that stone recurrence occurred significantly in elderly patients and the importance of PAD as a predictor. [6] A literature search indicated 11 studies describing PAD as a strong predictor of stone recurrence, [3, 6, 7, 11, 14, 17–22] while 3 works did not indicate such a significance. [4, 13, 23] Our study revealed PAD as a strong predictor of recurrence; although the relationship of PAD and stone recurrence remains unclear, Sugiyama et al. proposed the possibility of recurrence stone formation subsequently after bile juice reflux due to PAD[24].

Previous studies have implied that a particular type of PAD affects the rate of recurrence. Kim et al. and Sun et al. concluded that type 1 PAD poses a greater risk of BD stone recurrence compared with type 2 or 3 PAD. [18, 21] Oak et al. also mentioned that both type 1 and 2 PAD affected recurrence[14]. The mechanical pressure of PAD to the distal BD and its proximity to the major duodenal papilla proposedly disturbs bile flow and influences BD stone formation. [21, 25] In our study, the type 1 of PAD clearly correlates with recurrence.

Our study also showed multiple BD stones as predictor of recurrence. Oak et al. reported that the presence of multiple BD stones correlated with stone recurrence after endoscopic stone extraction [14], which is consistent with our findings. The exact correlation of stone recurrence and multiple BD stones remains unclear and warrants further investigation to discern if the bile microenvironment, such as the bacterial composition, affects the formation of multiple bile duct stones and stone recurrence.

To note, BD dilatation and the presence of calculous GB were indicated in several reports as predictors. [3–7, 10, 11, 13, 17, 22] We should bear in mind that other factors, especially above two factors may be significant depending on the patient landscape and context of the study.

Our study revealed that the recurrence rate did not clearly differ between the types of treatment for major duodenal papilla. However, EPLBD had a tendency to correlate with non-recurrence of BD stones, since no recurrence was observed in patients after EPLBD. Although studies with larger sample size are needed to confirm whether our findings are significant, Itokawa et al. prospectively analyzed 183 patients after endoscopic stone extraction with EPLBD (they added small ES before EPLBD) and noted that EPLBD was associated with a low rate of recurring BD stones[26]. They proposed minimal use of EML and the large bile duct orifice achieved by EPLBD led to less recurrence due to enhanced reduction of residual fragment stones compared with ES and EML.

There were limitation in our study. First, in our study, recurrence was defined as “symptomatic” BD stone re-formation with clinical signs. Therefore, there was the possibility of excluding patients with “asymptomatic” recurrent BD stones, which could change recurrence prevalence. Second, counting the stone number was sometimes difficult, especially in cases with muddy stone or large number of stones. Therefore, there was a possibility for slight miscounting of the number of stones. In addition, some patients lacked data and could not be used in our retrospective study.

In conclusion, PAD and multiple BD stones are strong predictors for BD stone recurrence after endoscopic stone extraction for naïve major duodenal papilla. EPLBD before stone extraction may correlate with non-recurrence of BD stones, however, too small number of EPLBD cases included to determine in this study. These results should be confirmed via a large prospective protocol.

### Ethics statement

All data and records in this study were accessed anonymously.
